# Rapid Solar-Light Driven Superior Photocatalytic Degradation of Methylene Blue Using MoS_2_-ZnO Heterostructure Nanorods Photocatalyst

**DOI:** 10.3390/ma11112254

**Published:** 2018-11-12

**Authors:** Ritika, Manjot Kaur, Ahmad Umar, Surinder Kumar Mehta, Surinder Singh, Sushil Kumar Kansal, H. Fouad, Othman Y. Alothman

**Affiliations:** 1 Dr. Shanti Swarup Bhatnagar University Institute of Chemical Engineering and Technology, Panjab University, Chandigarh 160014, India; ritikaabrol228@gmail.com (R.); sonubhinder@gmail.com (S.S.); 2 Department of Chemistry and Centre of Advanced Studies in Chemistry, Panjab University, Chandigarh 160014, India; manjotduggan@gmail.com (M.K.); skmehta@pu.ac.in (S.K.M.); 3 Department of Chemistry, Faculty of Science and Arts and Promising Centre for Sensors and Electronic Devices (PCSED), Najran University, Najran 11001, Saudi Arabia; 4 Department of Applied Medical Science, Riyadh Community College, King Saud University, Riyadh 11437, Saudi Arabia; menhfef@ksu.edu.sa or menhfefnew@hotmail.com; 5 Biomedical Engineering Department, Faculty of Engineering, Helwan University, Helwan 11792, Egypt; 6 Chemical Engineering Department, King Saud University, P.O. Box 800, Riyadh 11421, Saudi Arabia; othman@ksu.edu.sa

**Keywords:** MoS_2_-ZnO heterostructure nanorods, methylene blue dye, solar light irradiation

## Abstract

Herein, MoS_2_-ZnO heterostructure nanorods were hydrothermally synthesized and characterized in detail using several compositional, optical, and morphological techniques. The comprehensive characterizations show that the synthesized MoS_2_/ZnO heterostructure nanorods were composed of wurtzite hexagonal phase of ZnO and rhombohedral phase of MoS_2_. The synthesized MoS_2_/ZnO heterostructure nanorods were used as a potent photocatalyst for the decomposition of methylene blue (MB) dye under natural sunlight. The prepared MoS_2_/ZnO heterostructure nanorods exhibited ~97% removal of MB in the reaction time of 20 min with the catalyst amount of 0.15 g/L. The kinetic study revealed that the photocatalytic removal of MB was found to be in accordance with pseudo first-order reaction kinetics with an obtained rate constant of 0.16262 min^−1^. The tremendous photocatalytic performance of MoS_2_-ZnO heterostructure nanorods could be accredited to an effective charge transportation and inhibition in the recombination of photo-excited charge carriers at an interfacial heterojunction. The contribution of active species towards the decomposition of MB using MoS_2_-ZnO heterostructure nanorods was confirmed from scavenger study and terephthalic acid fluorescence technique.

## 1. Introduction

Water pollution is a grave environmental concern and perilous to the exquisite essence of the environment [1]. The release of noxious, persistent, carcinogenic, and mutagenic organic pollutants into the water bodies adversely affects the quality of water [2,3,4]. The contaminants discharged from numerous industries disturb the aquatic ecosystem by blocking the penetration of solar light into water, and thereby diminishing the photosynthetic activity of marine organisms [5,6,7,8]. Methylene blue (MB), a cationic synthetic dye, was invented by Caro in 1878 and it contains a heterocyclic aromatic structure with the molecular formula, C_16_H_18_N_3_SCl. It is extensively utilized for dyeing silk, paper, wood, and cotton. The acute inhalation of MB leads to troublesome effects on the human body; for instance, vomiting, irritation of the eyes, nausea, diarrhoea, cyanosis, jaundice, quadriplegia, dyspnea, tachycardia, methemoglobinemia, and tissue necrosis [9,10,11]. Therefore, it is of utmost importance to eradicate the venomous and persistent organic compounds from the wastewater [12,13,14,15]. Advanced oxidation processes, like photocatalysis, photo electro-Fenton, photo-Fenton, ozonation, sonolysis etc., are gaining increasing attention for the treatment of contaminated water as these have a tendency to convert recalcitrant organic pollutants into the least toxic compounds [16,17,18]. Among these methods, heterogeneous photocatalysis is widely used by the scientific community owing to its eco-friendly nature, cost effectiveness, and ability to completely mineralize the organic matter [19,20]. Keeping in view, various metal oxides/sulphides, like TiO_2_ [19], Fe_2_O_3_ [21], Bi_2_O_3_ [22], CdS [23], ZnS [24] etc., have been vastly explored as photosensitive heterogeneous catalysts for environmental remediation. Zinc oxide (ZnO) is an extensively studied semiconductor material owing to its peculiar characteristics, like high stability, good electron mobility, strong luminescence, inexpensive, high exciton binding energy, and non-toxicity [25,26,27,28]. However, rapid recombination of photogenerated charge carriers and low quantum efficiency inhibit its applications in the field of photocatalysis. These problems can be solved by using different approaches, such as coupling with other semiconductor materials or doping with non-metals/transition metal ions [29,30,31]. Two dimensional materials, like molybdenum disulphide (MoS_2_), have also attracted momentous attention due to its high absorption capacity, chemical inertness, easy availability, low toxicity, and thermal stability [32,33,34,35,36,37]. Various research groups have reported the integration of MoS_2_ and ZnO to form the desired heterostructures and further explored their potential applications in different fields. Zhang et al. fabricated MoS_2_-coated ZnO nanocomposite via hydrothermal method for the hydrogen production in the presence of simulated sunlight [38]. Zinc porphyrin dye-sensitized MoS_2_/ZnO heterostructure was synthesized by Yuan et al. and further employed in the generation of hydrogen under visible light [39]. Yan et al. hydrothermally prepared heterostructures based on nanoparticles of ZnO and nanosheets of MoS_2_, and utilized these for the sensing of ethanol [40]. Ze et al. prepared MoS_2_@ZnO quantum dots using a hydrothermal/chemical method, and explored the humidity sensing properties of the heterostructure [41]. Liu et al. have reported the growth of P-doped ZnO nanosheets decorated with an atomic MoS_2_ layer, and applied the prepared material for the sunlight driven photocatalytic application of MB dye [42]. The prepared P-doped ZnO nanosheets were grown on an Au-coated silicon substrate by chemical vapor transport and a condensation method at very high-temperatures of ~1000 °C. However, the MoS_2_ was decorated by the complex liquid exfoliation process with excess sonication and centrifugation methods. The growth of P-doped ZnO nanosheets decorated with an atomic MoS_2_ layer photocatalyst followed a very complicated, high-cost, rigorous, and high-temperature process [42]. It was observed that ZnO-MoS_2_ can be an efficient photocatalyst due to the fast charge transfer from the conduction bands (CBs) of ZnO and MoS_2_, which reduces the recombination of carriers, thus enhancing the reaction rate. The presence of MoS_2_ in the ZnO heterojunction structure can also enhance light adsorption in the visible range [42]. Therefore, due to the importance of ZnO/MoS_2_ heterojunction based photocatalysts, there is a need to develop a facile and simple method to prepare such catalysts, and, interestingly, we present here a facile, low-cost, and low-temperature method to synthesise high-quality and large-quantity ZnO-MoS_2_ heterostructure.

In the current study, MoS_2_-ZnO heterostructure nanorods were successfully synthesized and employed as a photocatalyst for the removal of MB dye under natural sunlight. The synthesized catalysts were extensively characterized by several techniques and are presented in the paper. Further, the impact of different operational reaction conditions, such as pH, catalyst amount, initial MB concentration, and scavengers, on the removal extent of MB has also been studied.

## 2. Materials and Methods

### 2.1. Synthesis and Characterizations of ZnO Nanoparticles and MoS_2_-ZnO Heterostructure Nanorods

**Reagents:** All the reagents were used as received without any purification. Zinc acetate dihydrate (Zn(CH_3_COO)_2_·2H_2_O, ≥98%), thiourea (CH_4_N_2_S, 99%), sodium hydroxide (NaOH, ≥97 %), potassium iodide (KI, ≥99%), formic acid (HCOOH, ≥98%), sodium chloride (NaCl, ≥99%), isopropanol (IPA; C_3_H_8_O, 99%) and methylene blue (C.I. 52015) were procured from Merck, Mumbai, India. Sodium molybdate dihydrate (Na_2_MoO_4_·2H_2_O, 99.5%) was purchased from Himedia, India. Ethanol (C_2_H_6_O, >99.9%) and terephthalic acid (TPA; C_8_H_6_O_4_, 98%) were obtained from Merck KGaA, Germany and Sigma Aldrich, respectively. The commercial photocatalyst TiO_2_ (PC-50) was procured from Millennium Inorganic Chemicals, Le Havre, France. 1M HCl and NaOH solutions were employed for the adjustment of pH using Mettler Toledo pH-meter (FEP 20).

**ZnO nanoparticles**: ZnO nanoparticles were hydrothermally synthesized using Zn(CH_3_COO)_2_·2H_2_O and NaOH. In the synthesis procedure, 0.25 M (5.487 g) of Zn(CH_3_COO)_2_·2H_2_O was added to 100 mL of double distilled water and continuously stirred for 30 min. Afterwards, a pH of 11 was maintained using 1 M NaOH solution and the suspension was poured into a Teflon-lined stainless steel autoclave kept at 100 °C for 15 h. The prepared ZnO was filtered and thorough washing was done with water/ethanol mixture and dried in an electric oven.

**MoS_2_-ZnO heterostructure nanorods**: MoS_2_-ZnO heterostructure nanorods synthesis was performed via the hydrothermal approach. For the preparation of MoS_2_/ZnO heterostructure nanorods, 4 mmol (0.967 g) of Na_2_MoO_4_·2H_2_O and 10 mmol (0.761 g) of CH_4_N_2_S were added separately to 35 mL of distilled water under continuous stirring. Consequently, 1.6 g of as-formed ZnO nanoparticles was dispersed in it and the suspension was magnetically stirred. After the stirring process, the suspension was transferred to Teflon-lined stainless steel autoclave for hydrothermal treatment, which was heated to 180 °C for 38.5 h. After completing the hydrothermal reaction, the vessel was cooled to room temperature and the obtained product was filtered and washed with alcoholic mixtures. The material was finally dried in an electric oven and then ground in order to obtain the fine powder.

**Characterizations:** The phase structure was determined by PANalytical X’Pert PRO diffractometer (Malvern Panalytical, Almelo, The Netherlands) and X-ray diffraction (XRD) patterns were obtained in the 2θ range from 20°–80° using Cu Kα radiations (λ = 1.54056 Å). The identification of functional groups was performed on a Nicolet iS50 FT-IR (Thermo Scientific, Waltham, MA, USA) spectrophotometer. The fluorescence (FL) spectrophotometer (Hitachi F-7000, Hitachi, Tokyo, Japan) was used for the measurement of FL emission spectrum at a room temperature. The diffuse reflectance spectrum (DRS) was taken on a UV-2600 (Shimadzu, Kyoto, Japan) UV-vis spectrophotometer using BaSO4 as a reference material. The general morphologies were observed by transmission electron microscope (TEM; Hitachi H-7500, Tokyo, Japan). The high resolution transmission electron microscope (HRTEM) and selected area electron diffraction pattern (SAED) of MoS2-ZnO heterostructure were analyzed by FEI Technai F20 microscope (FEI, Eindhoven, The Netherlands). The specific surface area was obtained from nitrogen adsorption-desorption isotherm using a Quantachrome Nova 2000e BET (Boynton Beach, FL, USA) and pore size distribution analyzer. The sample was pre-heated at 150 °C prior to BET measurements. The ultraviolet-visible (UV-vis) absorbance spectra were collected on a Systronics-2202 UV-vis spectrophotometer (Uvsar India, Ghaziabad, India).

### 2.2. Photocatalytic Degradation of MB Based on MoS_2_-ZnO Heterostructure Nanorods

The catalytic ability of the MoS_2_-ZnO heterostructure nanorods was explored for the decomposition of MB dye under natural sunlight. The light intensity of 65–75 Klux (Latitude 30°45′34″ N and Longitude 76°46′14″ E) was recorded on a CHY-332 digital light meter. For photocatalytic reactions, a specific amount of catalyst was dispersed into 100 mL of MB solution and kept in a dark environment under stirring for 30 min to achieve an adsorption/desorption equilibrium. Then, the solution was placed under solar light irradiation and aliquots (2 mL) were extracted from the beaker at specified time intervals. The catalyst was separated by filtering the solution through a 0.45 µm Chromafil syringe filter and the absorbance of the filtrate was monitored at λ_max_ = 664 nm on a UV-vis spectrophotometer. The degradation (%) was computed from Equation (1):

Degradation (%) = [(C_0_ − C)/C_0_] × 100 (1)

where, C_0_ is the MB concentration at the initial state and C is the MB concentration after illuminating with solar light at a specified time.

The role of reactive oxygen species in the removal of MB using MoS_2_/ZnO heterostructure nanorods was ascertained from a scavenger study. For this, 0.01 M of scavengers, such as HCOOH, KI, NaCl, and IPA, were added into the MB solution prior to the addition of the MoS_2_/ZnO heterostructure. The role of hydroxyl radicals (·OH) was validated using the terephthalic acid fluorescence technique. For the photocatalytic reaction, 5 × 10^−4^ M of TPA was added to 2 × 10^−3^ M NaOH solution. Then, 0.15 g/L of the synthesized catalyst, i.e., MoS_2_/ZnO heterostructure, was then dispersed into 100 mL of TPA solution and it was placed under solar light. At specified time periods, samples were collected, filtered through Chromafil syringe filter, and measured using FL spectrophotometer at λ_exc_ = 315 nm.

## 3. Results and Discussion

### 3.1. Characterization of As-Formed ZnO Nanoparticles and MoS_2_-ZnO Heterostructure Nanorods

**Figure 1:**
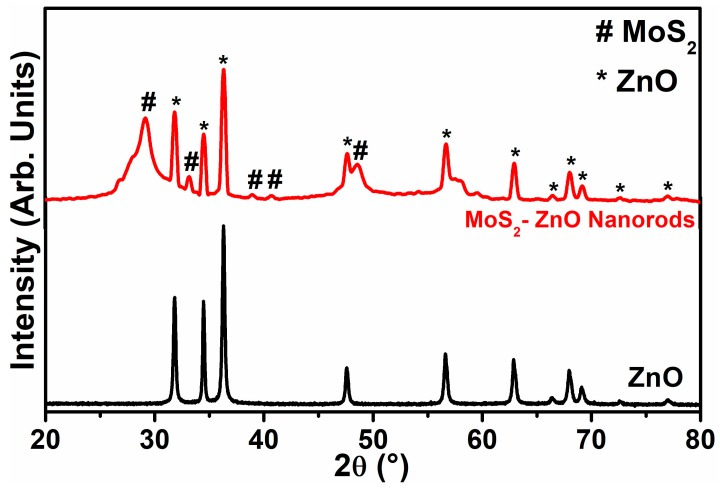
Typical XRD diffraction patterns of synthesized ZnO nanoparticles and MoS_2_-ZnO heterostructure nanorods.

The purity and structural characteristics of the synthesized materials were studied by the XRD technique. Figure 1 depicts the XRD diffractograms of pure ZnO nanoparticles and MoS_2_-ZnO heterostructure nanorods. Pure ZnO nanoparticles exhibited distinctive peaks appeared at 2θ = 31.8°, 34.4°, 36.2°, 47.6°, 56.6°, 62.8°, 66.3°, 67.9°, 69.1°, 72.6°, and 77.0° corresponded to (100), (002), (101), (102), (110), (103), (200), (112), (201), (004), and (202) crystallographic planes of ZnO, respectively. The XRD analysis of ZnO nanoparticles exhibited full consistency with the crystallographic planes of the wurtzite hexagonal phase of ZnO (Joint Committee on Powder Diffraction Standards) JCPDS card No. 36-1451) [43]. The typical XRD diffractograms of MoS_2_-ZnO heterostructure nanorods showed well defined peaks of ZnO along with MoS_2_ at 2θ = 29.1°,33.1°, 38.8°, 40.7°, and 48.5°, which can be ascribed to the (006), (101), (104), (015), and (107) lattice planes of the rhombohedral phase of MoS_2_, respectively. The XRD diffraction peaks of MoS_2_ are well matched with the reported JCPDS card No. 17-0744 and reported literature [44]. The XRD diffractogram of MoS_2_-ZnO heterostructure nanorods clearly revealed the existence of individual components of MoS_2_ and ZnO in the synthesized heterostructure nanorods. The crystallite size of ZnO and MoS_2_-ZnO heterostructure were computed from the most intense peak of the XRD pattern using Scherrer’s equation (Equation (2)):

D = 0.9 λ/βcosθ(2)

where, D is the crystallite size, λ refers to the wavelength of X-rays, β belongs to the full width at the half maxima value, and θ is considered as the Bragg’s angle. According to the above equation, the calculated crystallite size of pure ZnO nanoparticles and MoS_2_-ZnO heterostructure nanorods were ~38.45 nm and 33.52 nm, respectively.

**Figure 2:**
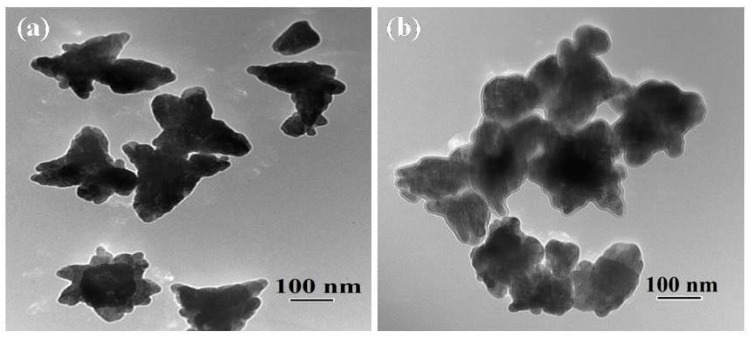
**(a,b)** Typical TEM images of pure ZnO nanoparticles.

**Figure 3:**
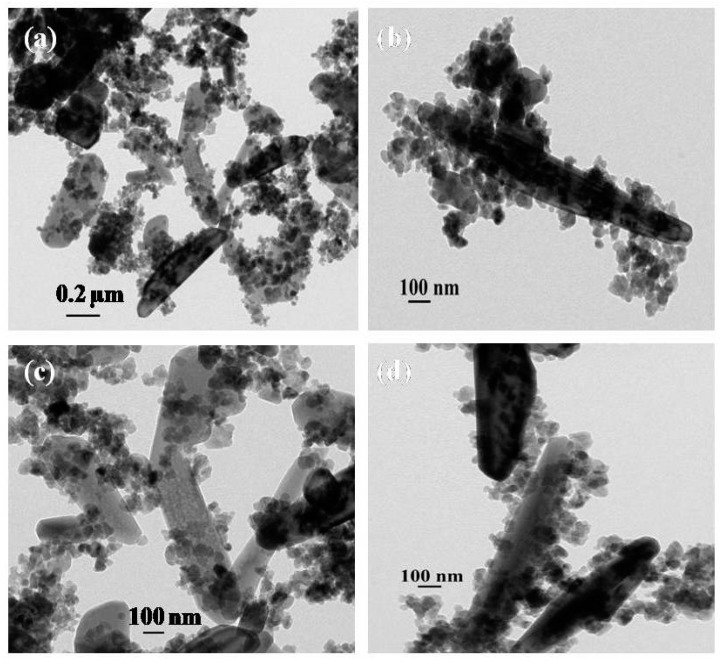
(**a**–**d**) Typical TEM images of prepared MoS_2_-ZnO heterostructure nanorods.

**Figure 4:**
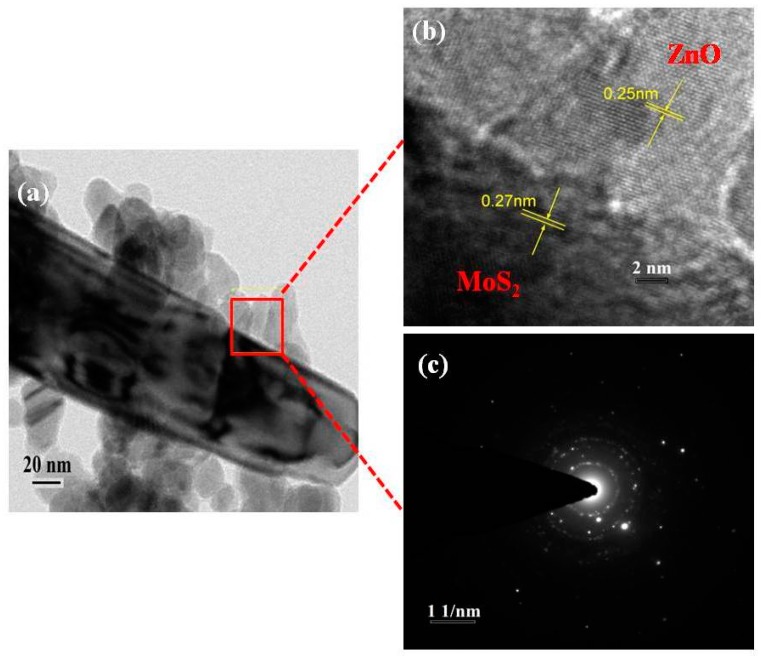
(**a**,**b**) HRTEM images and (**c**) SAED pattern of synthesized MoS_2_-ZnO heterostructure nanorods.

The morphological features of synthesized ZnO and MoS_2_-ZnO heterostructure were investigated using TEM. Figure 2a,b depicts the TEM micrographs of bare ZnO, which clearly demonstrate that the prepared sample possesses particle like morphologies, and, due to nanosize dimensions, it was termed as “nanoparticles”. Further, because of the dense growth, some agglomeration in the nanoparticles is also seen in the micrographs. Figure 3a–d shows the TEM images of synthesized MoS_2_-ZnO heterostructure nanorods. The TEM images clearly reveal the deposition of ZnO nanoparticles on the outer surfaces of the MoS_2_ nanorods. The synthesis of such MoS_2_-ZnO nanorods resulted in heterojunction formation, which is vital for the efficient charge transportation across the interface and leads to the high photocatalytic activity for the targeted dye. The high resolution transmission electron microscopy (HRTEM) images of the MoS_2_-ZnO heterostructure nanorods are shown in Figure 4a,b. The inter-planar spacing of 0.27 nm and 0.26 nm was observed for MoS_2_-ZnO heterostructure nanorods (Figure 4b). The lattice spacing of 0.27 nm can be indexed to the (101) crystallographic plane of pure MoS_2_ nanorods, however, the spacing of 0.25 nm matched well with that of the (101) plane of ZnO nanoparticles. The selected area electron diffraction (SAED) displayed a well-defined ring spot pattern, depicting the high crystallinity of the synthesized MoS_2_-ZnO heterostructure nanorods (Figure 4c). The HRTEM analysis confirmed the heterojunction formation between MoS_2_ and ZnO components and also showed full consistency with the obtained XRD results.

**Figure 5:**
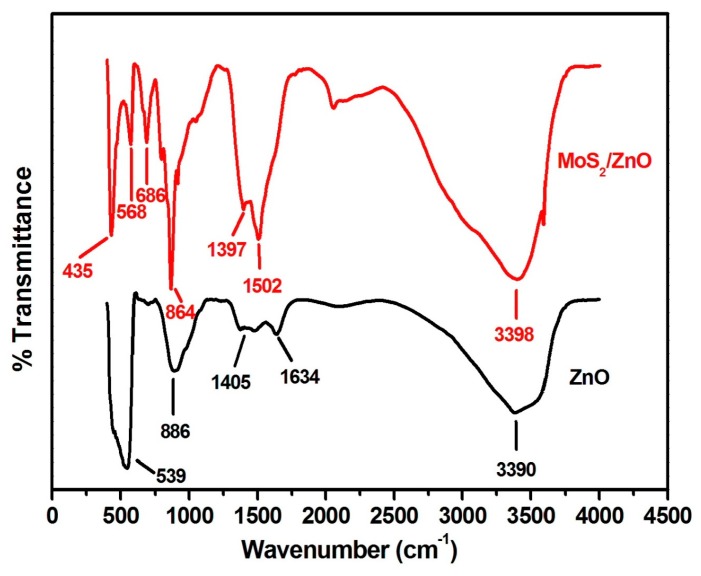
Typical FT-IR spectra of prepared ZnO nanoparticles and MoS_2_-ZnO heterostructure nanorods.

The presence of various functional groups was examined by FT-IR spectroscopy. Figure 5 displays the FT-IR patterns of bare ZnO nanoparticles and MoS_2_/ZnO heterostructures. The FT-IR pattern of ZnO nanoparticles displayed well defined absorption peaks at 539, 886, 1405, 1634, and 3390 cm^−1^. The characteristic peaks observed at 539 cm^−1^ and 886 cm^−1^ were due to the stretching and bending vibrating modes of Zn–O, respectively [45,46]. A peak at 1405 cm^−1^ might be accredited to the C=O bonding [47]. The broad peaks at 1634 cm^−1^ and 3390 cm^−1^ were related to the bending and stretching frequencies of the hydroxyl group, respectively [48,49]. The distinct FT-IR peaks of the MoS_2_/ZnO heterostructure were observed at 435, 568, 686, 864, 1397, 1502, and 3398 cm^−1^. A peak of Mo-S stretching vibration mode was found at 435 cm^−1^ [47,50]. A peak at 686 cm^−1^ might be related to the asymmetric vibration of the Mo-O group [51]. The peaks at 568 cm^−1^ and 864 cm^−1^ revealed the existence of stretching and bending vibration modes of Zn–O bond, respectively [46,49]. The bands observed at 1397 cm^−1^ and 1502 cm^−1^ corresponded to the C=O and C–O absorption, respectively [47,52]. A peak at 3398 cm^−1^ was due to the presence of surface bounded water molecules [49]. Thus, the FT-IR spectrum of MoS_2_/ZnO heterostructures indicated the successful coupling of MoS_2_ and ZnO in the prepared heterostructure.

**Figure 6:**
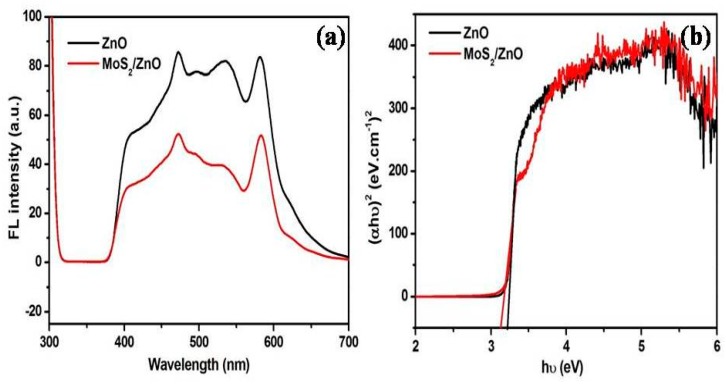
(**a**) Room temperature FL spectra and (**b**) Tauc’s plot of pure ZnO nanoparticles and MoS_2_/ZnO heterostructure nanorods.

The FL technique provides valuable information about the direct recombination of photoinduced charge carriers. Figure 6a illustrates the FL plots of ZnO nanoparticles and MoS_2_/ZnO heterostructure. Upon excitation at 290 nm, pure ZnO displayed emission peaks at 472 nm (blue-green band), 498 nm, and 533 nm (green band). An emission peak located at 472 nm was assigned to the band edge bound excitons [53]. The FL emission peaks at 498 nm and 533 nm were related to the defect emissions of ZnO, oxygen interstitials, and surface defects [38,49,54,55]. It was observed that FL emission intensity for the MoS_2_/ZnO heterostructure was remarkably quenched with respect to bare ZnO nanoparticles, depicting the reduction in the recombination rate of electron and hole pairs and thereby being responsible for the outstanding photocatalytic activity of the prepared MoS_2_/ZnO heterostructure.

The photo-absorption tendency of as-formed materials was explored using UV-vis DRS spectroscopy. The band gap was measured from the classical Tauc’s relation using Equation (3):

(αhν) = A(hν − E_bg_)^n^ (3)

where α, h, ν, A, and E_bg_ represent the absorption coefficient, Planck’s constant, frequency of light, proportionality constant, and energy band gap, respectively. The energy band gap was found to be 3.22 eV and 3.12 eV, respectively, for ZnO nanoparticles and the MoS_2_/ZnO heterostructure (Figure 6b). A decrease in the band gap was observed for the MoS_2_/ZnO heterostructure with respect to pure ZnO nanoparticles, indicating the better photo-absorption capacity of the prepared heterostructure.

**Figure 7:**
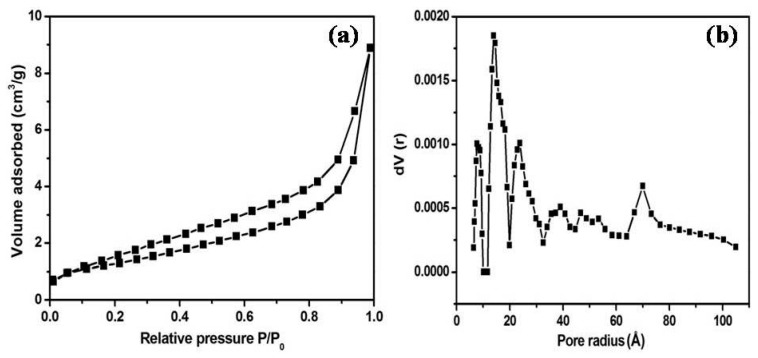
(**a**) Nitrogen adsorption-desorption isotherm and (**b**) DFT pore size distribution curve of MoS_2_/ZnO heterostructure nanorods.

The textural properties of the as-synthesized MoS_2_/ZnO heterostructure were studied using N_2_ adsorption/desorption isotherm and the pore size distribution (Figure 7a,b). The specific surface area and the total pore volume of the MoS_2_/ZnO heterostructure were estimated to be 8.132 m^2^/g and 6.590 × 10^−2^ cm^3^/g, respectively. The density functional theory (DFT) method was opted to find the average pore diameter and it was found to be around 2.74 nm for the synthesized MoS_2_/ZnO heterostructure.

### 3.2. Investigation of MoS_2_/ZnO Heterostructure for the Removal of MB under Natural Sunlight

**Figure 8:**
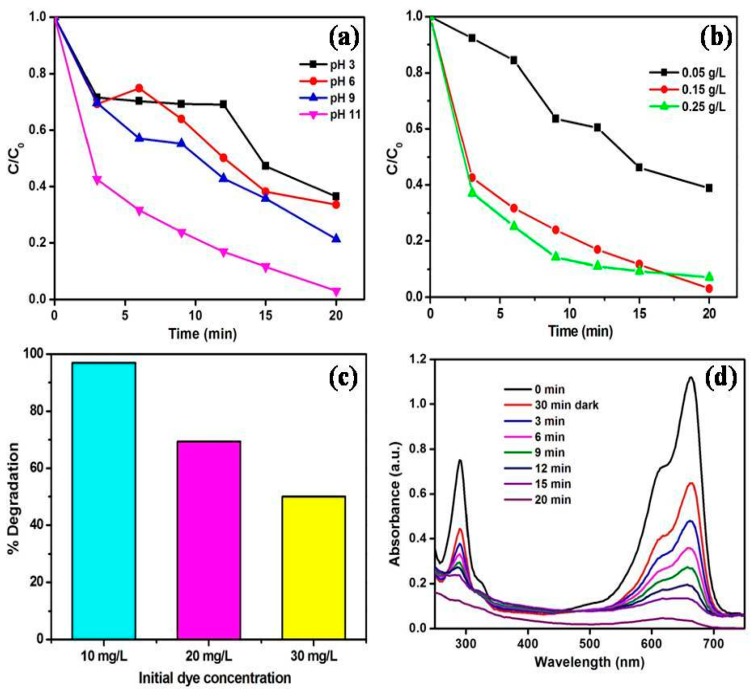
Effect of (**a**) pH; (**b**) catalyst amount; (**c**) initial methylene blue (MB) concentration towards the degradation of MB under solar light using MoS_2_/ZnO heterostructure and (**d**) time dependent UV-vis absorbance spectra of MB over the synthesized MoS_2_/ZnO heterostructure under solar light illumination (catalyst amount: 0.15 g/L, pH: 11, and MB concentration: 10 mg/L).

The catalytic performance of the synthesized MoS_2_/ZnO heterostructure was measured for the oxidation of MB dye under natural solar light. A chain of experiments was conducted to examine the impact of pH on the decomposition of MB by altering the pH from 3 to 11 using a catalyst dose of 0.15 g/L and substrate concentration of 10 mg/L as shown in Figure 8a. It was found that MB removal was enhanced from 63% to 66% with the increase in pH from 3 to 6. The removal efficiency was enhanced up to 78% after changing the pH to 9. The maximum decomposition rate for MB was found at pH 11 and about 97% decomposition of MB was acquired in the time period of 20 min. The impact of catalyst loading towards oxidation of MB was investigated by performing the reactions with different MoS_2_/ZnO heterostructure doses (pH 11 and dye concentration 10 mg/L). From Figure 8b, it was observed that on increasing the MoS_2_/ZnO heterostructure dose from 0.05 g/L to 0.15 g/L, the removal efficacy was enormously enhanced from 61% to 97%, owing to the existence of more reactive sites on the MoS_2_/ZnO heterostructure surface, resulting in the better adsorption of the dye molecules.

**Figure 9:**
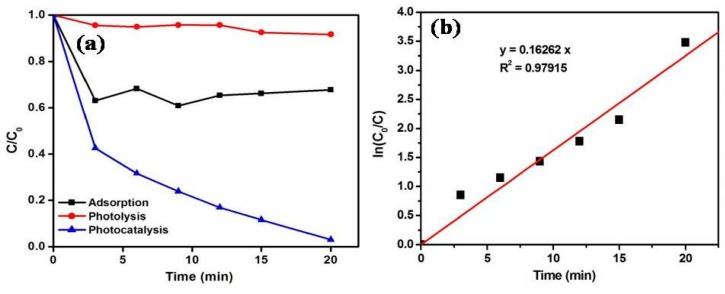
(**a**) Comparison between adsorption, photolysis, and photocatalysis for the removal of MB; (**b**) Kinetic curve for decomposition of MB over the prepared MoS_2_/ZnO heterostructure in the presence of solar light under optimized reaction parameters.

The degradation rate was decreased to 93% with increased catalyst loading up to 0.25 g/L, depicting the optimum MoS_2_/ZnO heterostructure amount to be 0.15 g/L for the catalytic reactions. The influence of the initial MB concentration towards the removal of MB was explored at optimized pH and catalyst dose conditions (Figure 8c). The removal rate of 97% was attained at 10 mg/L MB concentration. The degradation rates were diminished to 69% and 50% for 20 and 30 mg/L dye concentrations, respectively. Thus, from the series of experiments performed, it was clearly noticed that the maximum decomposition of MB was found at pH 11, catalyst amount of 0.15 g/L, and substrate concentration of 10 mg/L. Figure 8d depicts the UV-vis absorbance spectra for the oxidation of MB over the MoS_2_/ZnO heterostructure with respect to time (pH: 11, catalyst dose: 0.15 g/L, dye concentration: 10 mg/L). It was found that with the increase in illumination time, there was a reduction in the absorbance maximum (λ_max_ = 664 nm) of MB and 97% degradation was accomplished in 20 min. A blank experiment was performed under sunlight without the addition of the MoS_2_/ZnO heterostructure. The effect of photolysis is shown in Figure 9a and insignificant decomposition (8%) of MB was observed in the reaction time of 20 min. An adsorption experiment was performed with the addition of the MoS_2_/ZnO heterostructure without sunlight illumination and the obtained results are described in Figure 9a. Approximately, a 32% removal of MB was achieved using the MoS_2_/ZnO heterostructure. The kinetics of the reaction for the decomposition of MB over the MoS_2_/ZnO heterostructure was analyzed by a pseudo first-order kinetic model (Equation (4)):

ln(C_0_/C) = kt (4)

where C_0_ and C are the concentrations of MB before and after exposure to natural sunlight, k is the reaction rate constant acquired from the slope of the graph, and t is the irradiation time for the reaction. A graph between ln(C_0_/C) and t described that the oxidation of MB using the MoS_2_/ZnO heterostructure was found to be fitted well with the pseudo first-order reaction kinetics model, and a rate constant of 0.16262 min^−1^ was computed (Figure 9b).

**Figure 10:**
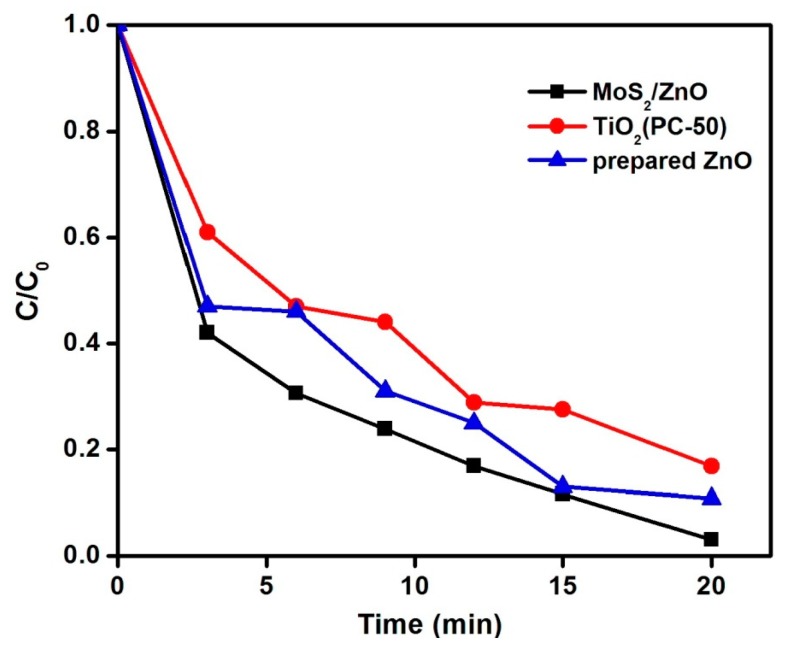
Comparison of MoS_2_/ZnO heterostructure, pure ZnO, and TiO_2_ PC-50 for the removal of MB under natural solar light (pH 11, catalyst dose: 0.15 g/L, and 10 mg/L aqueous MB solution).

The degradation extent of the MoS_2_/ZnO heterostructure was compared with pure ZnO nanoparticles and commercial TiO_2_ (PC-50) (Figure 10). The synthesized MoS_2_/ZnO heterostructure exhibited enhanced photocatalytic performance (97%) than pure ZnO (89%) and TiO_2_ PC-50 (83%) under identical reaction conditions.

### 3.3. Role of Reactive Oxygen Species for the Removal of MB Using MoS_2_/ZnO Heterostructure

**Figure 11:**
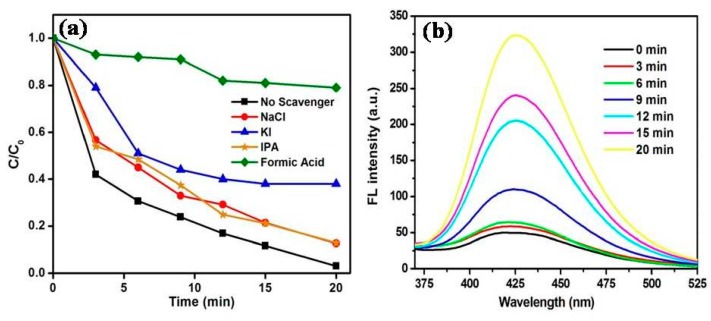
(**a**) Effect of scavengers on the photodegradation of MB over the MoS_2_/ZnO heterostructure and (**b**) fluorescence spectra of TPA over the synthesized MoS_2_/ZnO heterostructure under solar light irradiation.

Various quenchers were chosen to determine the role of active species involved in the photocatalytic decomposition of MB. Different quenchers, like KI (quencher for holes, h^+^, and surface bounded hydroxyl radicals, ·OH_s_), NaCl (quencher for h^+^), HCOOH (quencher for electrons, e^−^), and IPA (quencher for hydroxyl radicals, ·OH), were chosen to study their inhibitory effects. The results for the MB degradation using different scavengers over synthesized MoS_2_/ZnO photocatalyst under solar light are described in Figure 11a. It was found that the photocatalytic efficiency was reduced from 97% (without scavenger) to 16.8%, 86.8%, 87%, and 61.4% upon addition of HCOOH, NaCl, IPA, and KI, respectively, which verified the pivotal contribution of e^−^, h^+^, ·OH, and ·OH_s_ in the degradation process. The formation of ·OH during the course of the photocatalytic reaction was verified using the terephthalic acid fluorescence technique. The reaction of TPA with ·OH resulted in the generation of 2-hydroxyterephthalic acid (HTA), which is a highly fluorescent material, and showed an emission peak at around λ_max_ = 425 nm. The FL emission intensity of HTA is directly proportional to the formation of ·OH. As shown in Figure 11b, there was an enhancement in FL emission intensity with the progress in the photocatalytic reaction, verifying the pivotal role of ·OH towards the oxidation of MB dye [43].

### 3.4. Proposed Photocatalytic Degradation Mechanism

The conduction band (CB) and valence band (VB) potentials of MoS_2_ and ZnO can be calculated from Equations (5) and (6):

E_VB_ = X − E_e_ + 0.5 E_g_ (5)

**Figure 12:**
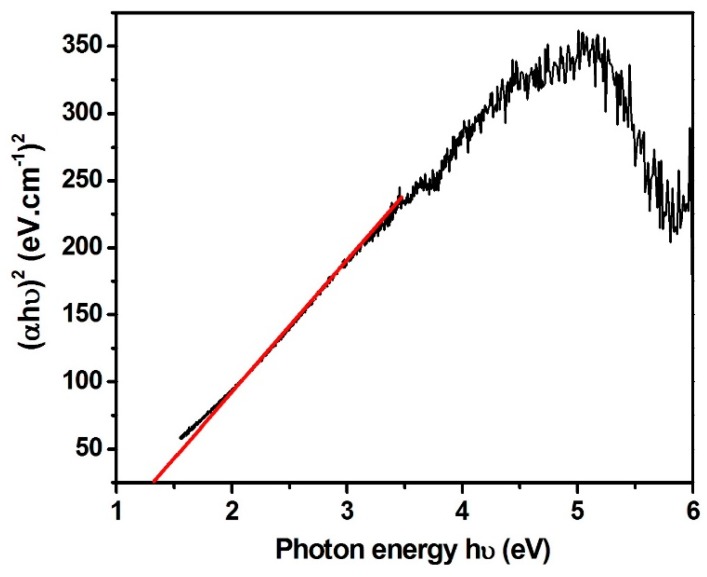
Plot between (αhν)^2^ and hν for pure MoS_2_.

E_CB_ = E_VB_ − E_g_ (6)

where X refers to the absolute electronegativity of the material; E_e_ represents the energy of free electrons on a hydrogen scale (~4.5 eV); and E_g_ corresponds to the band gap. The value of X for MoS_2_ and ZnO was reported to be 5.32 eV and 5.79 eV in the literature [56,57]. Figure 12 depicts the band gap of pure MoS_2_ and it was measured to be around 1.35 eV. The CB and VB potentials for MoS_2_ were measured to be 0.14 eV and 1.49 eV, respectively [58]. The corresponding CB and VB potentials for ZnO were found to be −0.32 eV and +2.9 eV, respectively.

**Figure 13:**
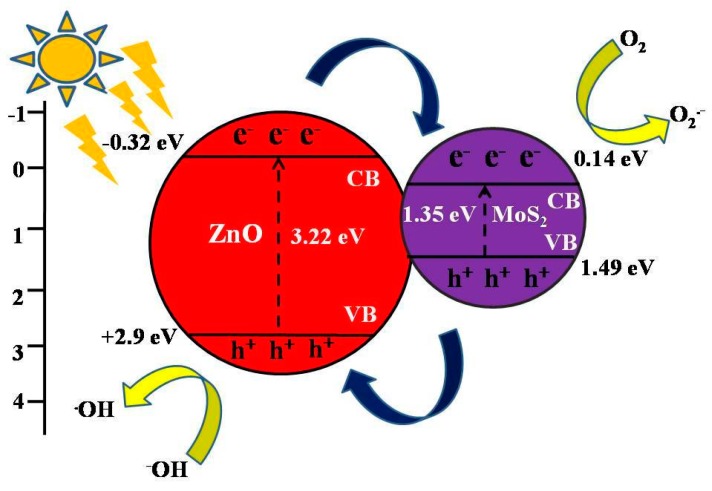
Pictorial representation of the mechanism for MB degradation using MoS_2_/ZnO heterostructure in the presence of natural solar light.

The plausible mechanism for the catalytic oxidation of MB over the MoS_2_/ZnO heterostructure under solar light is depicted in Figure 13. When sunlight was irradiated on the MoS_2_/ZnO heterostructure, both MoS_2_ and ZnO could be excited to yield e^−^/h^+^ pairs. The photogenerated e^−^ will shift from the CB of ZnO to the CB of MoS_2_ as the CB potential of ZnO is more negative than that of MoS_2_. Also, the photoinduced h^+^ will rapidly migrate from the VB of MoS_2_ to the VB of ZnO. The photoexcited e^−^ were captured by adsorbed molecular oxygen (O_2_) to yield superoxide anion radicals (O_2_^−^). MoS_2_ can capture e^−^ due to its high conductivity, and thus suppressed the recombination rate of photoexcited charge carriers [59,60]. The photo-generated h^+^ also reacted with OH^−^ or H_2_O, resulting in the production of **·**OH, which are accountable for the decomposition of noxious organic contaminants. Thus, the prepared MoS_2_/ZnO heterostructure was utilized as a potent catalyst for the decomposition of MB under natural solar light. Different oxidation and reduction reactions (Equations (7)–(10)) that occurred over the MoS_2_/ZnO heterostructure surface under natural sunlight irradiation for the generation of reactive species are given below:

ZnO + hν → e^−^_CB_ + h^+^_VB_ (7)

MoS_2_ (e^−^_CB_) + O_2_ → O_2_^−^ (8)

ZnO (h^+^_VB_) + OH^−^ →·OH (9)

·OH + MB → CO_2_ + H_2_O + simpler molecules (10)

Table 1 demonstrates the comparison of the MoS_2_/ZnO heterostructure for the decomposition of various pollutants under different light sources and it could be inferred that the present work offered excellent catalytic behavior for the removal of harmful organic contaminants.

## 4. Conclusions

In summary, MoS_2_/ZnO heterostructure was prepared through a facile hydrothermal route and extensively characterized in detail by spectroscopic and analytical techniques. The synthesized MoS_2_/ZnO heterostructure showed excellent catalytic behavior for the decomposition of MB under natural solar light. Approximately, 97% decomposition of MB was obtained at pH 11 with a catalyst dose of 0.15 g/L. The FL intensity of MoS_2_/ZnO heterostructure was strongly quenched as compared to pure ZnO, thereby increasing the photochemical quantum efficiency of the heterostructure. The superior photocatalytic efficacy of the MoS_2_/ZnO heterostructure could be assigned to the effective charge transportation of photoinduced e^−^/h^+^ pairs. The present study demonstrated that the MoS_2_/ZnO heterostructure can be employed as a marvelous photocatalytic material for the deterioration of noxious contaminants in terms of environmental restoration.

## Figures and Tables

**Table 1:** Comparison of photocatalytic efficiency of MoS_2_/ZnOheterostructures for the degradation of pollutants in the presence of different light sources.

Catalyst	Target Pollutant	Light Source	Reaction Time	Degradation (%)	Reference
MoS_2_-RGO doped ZnO (1 wt % of MoS_2_-RGO in ZnO)	MB and carbendazim	Natural solar light	60 min	98% for MB and 97% for carbendazim	[50]
MoS_2_/ZnO	Rhodamine B	Simulated solar light	90 min	91.4%	[61]
MoS_2_/ZnO	Phenol red	UV and visible light	50 min under UV light	93%	[62]
			80 min under visible light	90%	
P-doped ZnO nanosheets decorated MoS_2_	MB	Natural solar light	6 min for MB	95%	[42]
ZnO-g-C_3_N_4_ (50%)/MoS_2_ (1%)	MB and atrazine	UV-visible light	30 min for MB	99.5% for MB	[63]
			300 min for atrazine	84.9% for atrazine	
**MoS_2_/ZnO**	**MB**	**Natural solar light**	**20 min**	**97%**	**This work**
